# Transverse myelitis and acute HIV infection: a case report

**DOI:** 10.1186/1471-2334-14-149

**Published:** 2014-03-19

**Authors:** Paulo Andrade, Cristóvão Figueiredo, Cláudia Carvalho, Lurdes Santos, António Sarmento

**Affiliations:** 1 Department of Infectious Diseases, Hospital de São João and University of Porto Medical School, Porto, Portugal

**Keywords:** Transverse myelitis, HIV, Acute infection

## Abstract

**Background:**

Most HIV infected patients will develop some sort of neurologic involvement of the disease throughout their lives, usually in advanced stages. Neurologic symptoms may occur in acute HIV infection but myelopathy in this setting is rare. Up until this date, only two cases of transverse myelitis as a manifestation of acute HIV infection have been reported in the literature. Therapeutic approach in these patients is not well defined.

**Case presentation:**

A 35 year-old male Caucasian recently returned from the tropics presented to our hospital with urinary retention and acute paraparesis. After extensive diagnostic workup he was diagnosed with acute HIV infection presenting as transverse myelitis. Full neurologic recovery was observed without the use of anti-retroviral therapy.

**Conclusion:**

Acute spinal cord disorders are challenging, as they present a wide array of differential diagnosis and may lead to devastating sequelae. Timely and rigorous diagnostic workup is of the utmost importance when managing these cases. Clinicians should be aware of the protean manifestations of acute HIV infection, including central nervous system involvement, and have a low threshold for HIV screening.

## Background

HIV-associated neurological syndromes are diverse and usually diagnosed at advanced stages of the disease [1]. However, neurological involvement can also occur at early stages of the infection, making the diagnosis challenging [2-4]. In tropical areas the differential diagnosis of neurological disorders is particularly difficult, as a broad range of infectious causes need to be considered. We report a case of an acute HIV infection presenting as transverse myelitis in a patient returning from sub-Saharan Africa. The diagnostic approaches of acute spinal cord disorders are also reviewed.

## Case presentation

A 35-year-old Caucasian male presented to an Emergency Room of a Portuguese tertiary care hospital with urinary retention and diminished strength in the lower limbs. He had returned a week before from Angola where he had been working as a construction worker for the past 10 months (Table 1). He had been vaccinated for yellow fever, poliomyelitis, typhoid fever and meningococcal disease in advance. Excluding an episode of malaria soon after his arrival to Angola, he was asymptomatic until 6 weeks before returning home to Portugal, when he and other co-workers had an acute onset of fever, vomiting and diarrhoea after sharing a meal. Over the following days, while he was still recovering, he woke up suddenly with pain in the umbilical area where he noticed three red spotted lesions that became enlarged and necrotic. He was told by a medical doctor that he had most likely been bitten by a spider and was treated with local dressings and analgesia, with improvement. One week later he developed fever, non productive cough, progressive dyspnoea and thoracic pain that he described as bilateral, oppressive and “belt-like”. Additionally, generalised myalgia and fatigue gradually installed. He was treated in a local clinic with antibiotics without resolution of symptoms. Unable to work, he returned to Portugal and visited another hospital in the same day for persisting fatigue and thoracic pain. Inflammatory parameters were not elevated and chest roentgenography was normal. He was sent home on analgesics. Six days later he presented to our tertiary care hospital complaining of worsening fatigue, thoracic pain and urinary retention.

**Table 1:** Timeline of the main clinical events

**PRIOR TO HOSPITAL ADMISSION**	
**Week 1**	Travels to Angola.
**Week 4**	Uncomplicated malaria episode.
**Week 32**	Ear piercing.
**Week 34**	Probable gastroenteritis and arthropod bite.
**Week 35**	Fatigue, fever, myalgia, non-productive cough, thoracic pain (belt-like).
	Treated with antibiotics.
**Week 40**	Persisting fatigue and thoracic pain.
	Returns to Portugal and seeks medical care. Discharged on analgesics.
**HOSPITAL ADMISSION**	
**Day 1**	Worsening fatigue and thoracic pain.
	Urinary retention, paraparesia and hypoaesthesia.
	HIV screening positive, Inno-Lia™ indeterminate.
	MRI suggestive of myelitis.
	Ceftriaxone, gancyclovir and methylprednisolone.
**Day 3**	Worsening neurologic symptoms.
	Mild CSF pleocytosis and protein elevation.
	Doxycicline and human immunoglobulin. Stops gancyclovir.
**Day 10**	Progressive and complete resolution of symptoms.
	HIV-1 RNA detection on blood and CSF. 760/mm^3^ T-CD4 count.
**Day 21**	HIV screening positive, Inno-Lia™ indeterminate.
	Discharged home.
**OUTPATIENT CLINIC**	
**Month 3**	Assymptomatic.
	Inno-Lia™ positive.
**Month 12**	Assymptomatic.
	Progressive decline of T-CD4 count to below 400/mm^3^.
	Anti-retroviral therapy instituted.

CSF: cerebrospinal fluid; MRI: magnetic resonance imaging.

Past medical history was irrelevant. The patient denied unprotected sexual contacts in the past ten months, intravenous drug use, blood transfusions or past surgical procedures. He had performed an ear piercing two months before in Angola.

On admission the patient was afebrile. Blood pressure was 124/68 mmHg, his pulse was regular with 80 beats per minute and respirations 14 breaths per minute. Neurological examination revealed diminished motor strength in lower extremities (grade 3/5), reduced tactile and pain sensations below L1, normal deep tendon reflexes and bilateral indifferent plantar responses. No cognitive dysfunction or neck rigidity was noticed. Ophthalmologic and cranial nerves examination was normal. A periumbilical triangular scar was observed, with no inflammatory signs. The remainder of physical examination was unremarkable.

**Figure 1:**
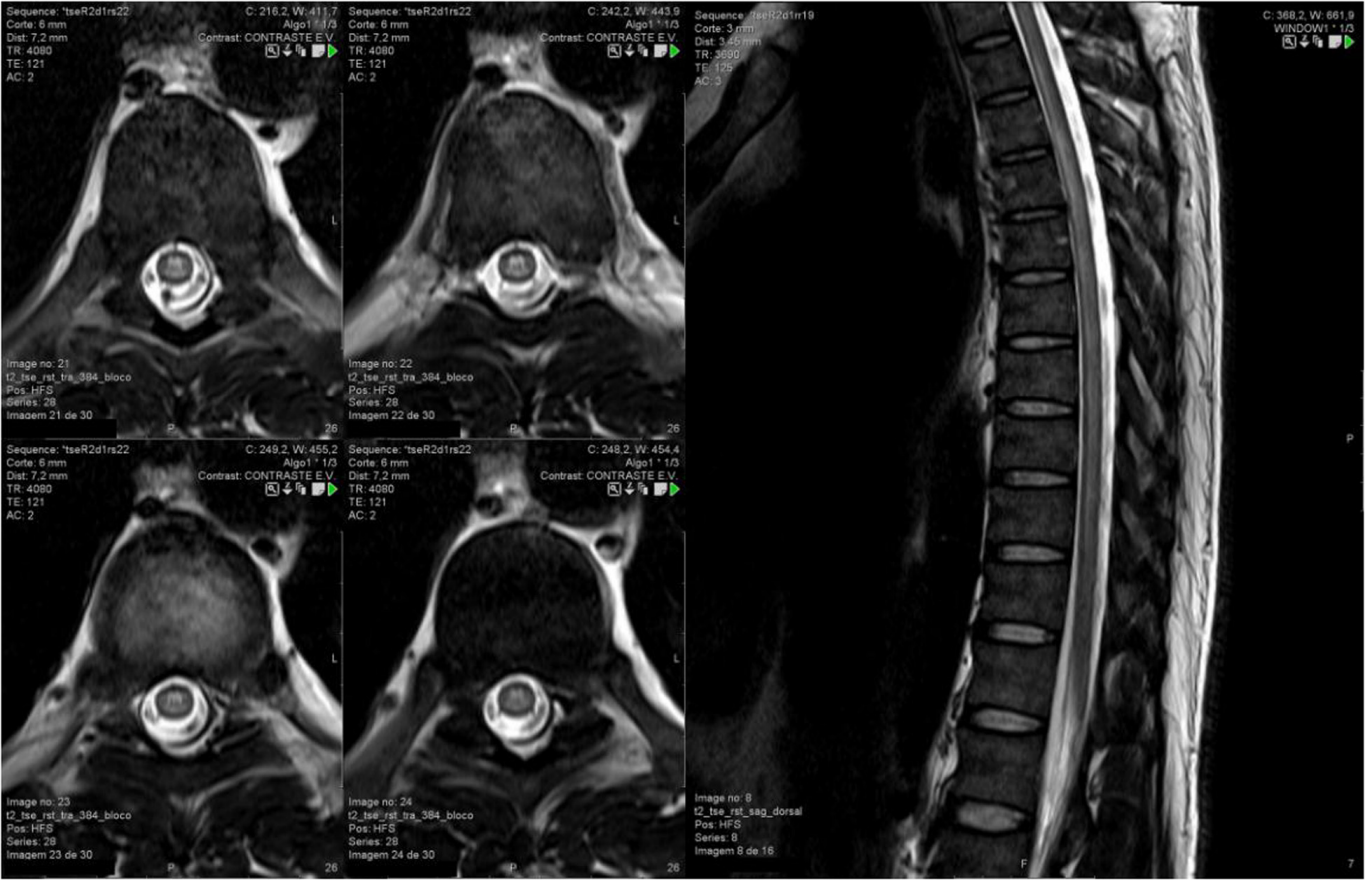
**Axial and sagittal T2-weighted magnetic resonance images.** Bilateral and symmetrical T2 hyperintensity in the thoracic spinal cord, extending from D2 to D11, predominantly involving the posterior columns. No swelling, atrophy or enhancement were present. Although not specific, these findings might be consistent with HIV myelopathy.

Routine laboratory data was normal, including complete blood count (Hb 15.6 g/dL, WBC 9.07 × 10^9^/L with 81% neutrophils, platelets 325 × 10^9^/L), renal and liver function tests. Thick blood smear and rapid diagnostic test for malaria were negative. HIV test (4th generation ELISA) was positive and Inno-Lia™ test was indeterminate (positive gp41 band). Chest roentgenography was normal. Cerebrospinal fluid (CSF) examination revealed 302 leukocytes/μL, glucose 0.56 g/L and protein content 0.74 g/L. Gram stain was negative. Magnetic Resonance Imaging (MRI) of the brain was normal, but MRI of the medulla showed extensive hyperintense signal in the long TR sequence throughout C4 to T11, especially in the posterior columns, suggesting inflammatory/infectious aetiology (Figure 1).

He was admitted with a diagnosis of acute transverse myelitis and treatment with ceftriaxone (2 g IV bid), ganciclovir (400 mg IV tid) and methylprednisolone (1 g IV qd for 3 days and then tapered to 1 mg/Kg/d) was started.

Over the two following days his clinical condition deteriorated, with intensifying upper thoracic pain, progressing hypoaesthesia up to T10 and dysaesthesia becoming more disabling and characterised by numbness and ice cold sensation. Constipation and urinary retention were also present.

A second lumbar puncture performed at day 3 revealed 15 leucocytes/μL, glucose 0.98 g/L and protein content 0.86 g/L. India ink stain was negative.

Doxycicline (100 mg bid) and human immunoglobulin (30 gr IV qd) were associated at this point. Ganciclovir was discontinued after negative CMV results (Table 1).

Significant improvement was observed within the next days and one week later the patient was able to walk unassisted, with complete resolution of dysaesthesia, urinary retention and constipation. Three weeks after admission he was discharged home asymptomatic, having completed 21d of ceftriaxone, 7d of methylprednisolone, 7d of doxycicline and 5d of human immunoglobulin.

HIV testing was repeated before discharge: 4th generation ELISA was positive and Inno-Lia™ remained indeterminate (positive gp41 band). All cultures were negative, as well as several serology and PCR-based technique tests (Table 2). Convalescent titters were repeated for *Borrelia*, *Rickettsia conorii*, West Nile Virus and Dengue, all negative. HIV-1, subtype K RNA copies were quantified in blood and CSF: 743.000 and 77.700 cp/mL respectively. By the time these results were known the patient was already improving significantly and no antiretroviral therapy was started. His CD4 count was 408/mm^3^ on admission and 760/mm^3^ in a second determination before discharge.

**Table 2:** Main laboratory results for diagnostic workup

**Biological specimen**	**Culture**	**PCR techniques**	**Serology**	**Others**
CSF	Bacteria, fungi and mycobacteria all negative	HSV 1&2, H-6, CMV, EBV, VZV, Enterovirus, WNV, JCV, HTLV1, *Rickettsia* spp, *Borrelia* spp, *M. pneumoniae*, *L. monocytogenes*, MTC all negative	VDRL, FTA-ABS all negative	Malignant cells negative, immunoelectrophoresis normal, India ink stain negative
		HIV-1 (77.700 cp/mL)		
Blood	Bacteria and fungi all negative	CMV negative	VDRL, TPPA/TP, *Borrelia* spp, *Rickettsia conorii*, HSV 1&2, EBV, Dengue, WNV all negative	CMV antigen negative
		HIV-1 (743.000 cp/mL)	CMV, *T. gondii* (IgM negative, IgG positive)	T-CD4 cell count (408/mm^3^)
			HIV (ELISA positive, Inno-Lia™ indeterminate with positive gp41 band)	

CMV: cytomegalovirus; CSF: cerebrospinal fluid; EBV: Epstein-Barr virus; H-6: Herpes 6 virus; HSV: Herpes simplex virus; HTLV1: human T lymphotropic virus Type 1; JCV: JC virus; MTC: Mycobacterium tuberculosis complex; PCR: polymerase chain reaction; VZV: Varicella-Zoster virus; WNV: West Nile Virus.

Three months later the patient presented to the outpatient clinic and was asymptomatic. HIV Inno-Lia™ test was then positive (positive p17, p24, p31, gp41 and gp120). He repeated a medullar MRI 8 weeks after discharge: previous findings had resolved completely and no pathological changes were found.

After one year of follow up he continued asymptomatic but with a CD4 count decline to below 400/mm^3^ his viral load had stabilized around 130.000 cp/mL. He started highly active anti-retroviral therapy (HAART) with abacavir/lamivudine and efavirenz, and 6 months later he had undetectable viral load with increased CD4+ cell count.

## Conclusion

Differential diagnosis of an evolving myelopathy is broad and might include compressive, vascular, immune-mediated and infectious aetiologies. Initial evaluation with appropriate imaging should focus on excluding compressive aetiology for which urgent neurosurgical evaluation is mandatory [5]. MRI with gadolinium contrast agent should therefore be obtained in a short-time period. If no compressive cause is identified, a lumbar puncture should be performed to distinguish an inflammatory from a non-inflammatory condition (such as ischemia, epidural lipomatosis or fibrocartilagineous embolism). Precipitous onset of symptoms (usually reaching maximal severity in < 4 h), absence of pleocytosis in CSF and no gadolinium enhancement on spinal cord MRI are highly suggestive of vascular causes [6]. If an inflammatory myelopathy is identified further evaluation should be undertaken. Clinical features, laboratory studies and imaging findings provide useful information for neurological conditions such as multiple sclerosis, neuromyelitis optica and systemic inflammatory diseases such as systemic lupus erythematosus, sarcoidosis and Sjögren Syndrome [6]. Nonetheless, infections are thought to be responsible for most cases of myelitis. Virus, bacteria, fungi or parasites might cause central nervous system (CNS) injury, either due to direct cytolytic effects or through secondary immune mediated processes. Acute disseminated encephalomyelitis (ADEM) is a monophasic inflammatory demyelinating disorder that can follow an infectious disease or vaccination. Unlike Guillain-Barré syndrome, that affects peripheral nerves, ADEM involves the CNS and therefore should also be considered [6].

Treatment of myelitis should be tailored to the individual patient. Antibiotics, antivirals, high doses of corticosteroids and intravenous immunoglobulin have been used, but evidence on the efficacy of most strategies is lacking.

Our patient presented several diagnostic challenges. Compressive causes had been excluded by early MRI. Acute transverse myelitis (ATM) diagnosis was supported by acute paraparesis with bilateral sensory findings, impaired sphincter function, the presence of spinal segmental level of sensory disturbance and the evidence of inflammation within the spinal cord demonstrated by CSF pleocytosis. Given the wide range of possible causes for ATM and taking into account the particular details of our patient history we were confronted with a wide range of challenging hypothesis: are we facing post-infectious myelitis secondary to acute gastroenteritis? Is this a toxin mediated myelitis caused by spider or snake venom? What if the periumbilical skin lesion is a tick bite sequelae, and *Borrelia* or *Rickettsia sp* are implicated? What about viral myelitis, the most common aetiology for ATM? Shouldn’t we consider viruses that can cause myelitis worldwide (Herpes viruses, Enterovirus) but also those than can be present in Africa (Dengue, West Nile virus, HTLV1)? Finally, how should we value a positive HIV screening test when no high risk exposure is identified? Could it be a false positive in the setting of another viral illness? Or could myelitis be the presentation of an acute retroviral syndrome?

It is estimated that in 40 to 70 percent of HIV-1–infected people neurologic complications will develop [7]. Viral infection can directly affect the central and peripheral nervous systems or lead to increased susceptibility to opportunistic infections due to immunodeficiency [8]. Direct CNS involvement most commonly presents as a HIV encephalitis, HIV leucoencephalopathy, vacuolar leucoencephalopathy or vacuolar myelopathy. CNS opportunistic infections to be considered include toxoplasmosis, cryptococcosis, tuberculosis and viral encephalitis such as CMV, among others [9]. Although less common, neurological manifestations can occur at early stages of HIV infection. These include peripheral neuropathy, radiculopathy, facial palsy and Guillain-Barré syndrome [2-4]. In rare cases, CNS may be predominantly affected, and acute or subacute transverse myelitis may be the presenting manifestation of HIV. Overall, ATM is an uncommon disease, with an estimated incidence of 3.1 cases per 100.000 patient years [10]. Classic symptomatic acute HIV infection is characterized by a mononucleosis-like syndrome, with fever, lymph node enlargement, pharyngitis, maculo-papular rash, myalgia an arthralgia; thrombocytopenia and leukopenia are common laboratory findings. In this setting, ATM is exceedingly rare, with only two cases previously reported [11,12]. Steroids and immunoglobulin are usually recommended for treatment and in the context of retroviral infection ARV therapy is likely beneficial, based on theoretical grounds. In one reported case, in the pre-HAART era, partial improvement with neurologic sequelae was observed [11]. In another, HAART and corticosteroids seemed beneficial, ensuring full recovery [12]. In our particular case, HIV infection wasn’t the most likely diagnosis at admission, given the absence of previous high risk exposure and the presence of more plausible alternative hypothesis. Therefore, the patient received a combination of anti-inflammatory and antimicrobial drugs and complete clinical and imagiological resolution was documented. The fact that by the time HIV infection was confirmed the patient was already recovering supported our decision of not starting ARV then.

We described a very rare case of acute HIV infection presenting as ATM in a patient where the suspicion index for HIV infection was rather low. The long list of alternative causes led to a particularly elusive diagnosis. Diagnosis of transverse myelitis following acute HIV infection was made after excluding other aetiologies. Our patient also illustrates a unique case of complete recovery without HAART. This report highlights the large spectrum of clinical presentations an acute HIV infection might have and the need for a low threshold for HIV screening.

## Consent

Written informed consent was obtained from the patient for publication of this Case report. A copy of the written consent is available for review by the Editor of this journal.

## Abbreviations

(MRI): Magnetic resonance imaging; (HAART): Highly active anti-retroviral therapy; (CNS): Central nervous system; (ADEM): Acute disseminated encephalomyelitis; (ATM): Acute transverse myelitis.

## Competing interests

The authors declare that they have no competing interests.

## Authors’ contributions

PA, CF and CC were responsible for the patient’s management during hospitalization and follow-up after discharge, have collected all significant clinical information and drafted this manuscript. LS and AS have reviewed, redrafted and given significant contribution to the final version. All authors have given final approval of this version.

## Pre-publication history

The pre-publication history for this paper can be accessed here:

http://www.biomedcentral.com/1471-2334/14/149/prepub
